# Physical impairments and physical therapy services for minority and low-income breast cancer survivors

**DOI:** 10.1186/s40064-016-2455-3

**Published:** 2016-08-02

**Authors:** Ann Marie Flores, Jason Nelson, Lee Sowles, Karen Bienenstock, William J. Blot

**Affiliations:** 1 Department of Physical Therapy, Movement and Rehabilitation Sciences, Center for Cancer Survivorship Studies (CCSL), Northeastern University, Boston, MA USA; 2 Biostatistics Research Center, Institute for Clinical Research and Health Policy Studies, Tufts University Medical Center, Boston, MA USA; 3 Office of Health Equity, Maine Center for Disease Control and Prevention, Augusta, ME USA; 4 International Epidemiology Institute, Rockville, MD USA; 5 Vanderbilt-Ingram Cancer Center, Vanderbilt University Medical Center, Nashville, TN USA

**Keywords:** Impairments, Income, Race, Physical therapy, Breast cancer

## Abstract

**Purpose:**

We describe impairments after breast cancer and its treatment for African American (AA), non-Hispanic white and low-income breast cancer survivors (BCS) and whether physical therapy (PT) was utilized to address these impairments.

**Methods:**

BCS from the Southern Community Cohort Study (SCCS) were surveyed about self-reported BC treatment-related impairments (shoulder impairment, muscle weakness, pain, fatigue, skin numbness, abnormal posture) and referral to PT for impairments. We compared impairments by race, income and PT utilization. We used a cross-sectional design.

**Results:**

Among 528 BCS interviewed (266 whites; 262 AA), mean age 64, those with low incomes were more likely to report muscle weakness, pain and postural abnormalities, and a greater total number of impairments than those with higher incomes. Racial differences were few. PT utilization tended to be low, with AAs more likely than whites to utilize PT if they had shoulder impairment or pain, whereas no monotonic trends across income levels were seen in PT utilization.

**Conclusions:**

Low-income level was associated with greater prevalence of BC-related physical impairments, but not higher PT utilization. There appears to be a possible under-utilization of PT, particularly for those with low incomes.

## Background and significance

Side effects such as physical impairments (impairments) are persistent reminders of the breast cancer (BC) experience (Hewitt et al. 2006). The most common breast cancer (BC)—related impairments are lymphedema, pain and fatigue. These and less studied impairments such as upper extremity muscle weakness, loss of range of motion, altered skin sensation and integrity, and abnormal posture and shoulder movement (Hewitt et al. 2006; Battaglini et al. 2014; Binkley et al. 2012) fall under the scope of physical therapy (PT) practice (American Physical Therapy Association 2001). Impairment prevalence varies widely. Impaired shoulder range of motion affects up to 67 % of BC survivors (BCS); arm weakness affects 9–28 % (McNeely et al. 2010; Lee et al. 2007); shoulder/arm pain affects 9–68 % (McNeely et al. 2010); and 26 % report having difficulty with activities of daily living (Voogd et al. 2003). Fatigue affects 90 % of all cancer survivors (Cramp and Byron-Daniel 2012). None of these studies, however, consider differences by race.

African American (AA) women have higher BC burden than non-Hispanic whites (“whites”) (Tannenbaum et al. 2013), yet studies on impairments have mostly white middle-income women with health insurance and do not consider combined effects of race and income. Socioeconomic status and race have strong explanatory effects on cancer mortality (Tian et al. 2012), but the relationship of impairment prevalence and PT utilization are less clear.

Rehabilitation such as PT is recommended as part of BC care but it is not part of standard care planning (Alfano et al. 2012). We examine the associations between race and income with BC-related impairments and whether PT is utilized to treat impairments.

## Methods

### Study participants

Our cross-sectional study includes female BCS enrolled in the Southern Community Cohort Study (SCCS), a long-term, prospective, population-based cohort study that recruited 85,000 adults (age 40–79)—two-thirds AA—from twelve southern states during 2002–2009. Eligibility criteria required participants to speak English and not diagnosed or treated for cancer within the preceding year prior to cohort entry. Most SCCS participants were recruited from community health centers serving low-income individuals in medically underserved areas with face-to-face interviews for survey data collection. SCCS recruitment and survey methods are described online at www.southerncommunitystudy.org (Signorello et al. 2005, 2010).

After institutional review board approval (Vanderbilt University and Northeastern University), we obtained contact information for women in the SCCS who were still alive by September 2010 and a BC history. Contact attempts were made through 2012 with a recruitment packet that included an introduction letter, consent document, and a set of answer choices (similar to hand cards for face-to-face interviews). We made up to 15 follow-up telephone calls to schedule and conduct the study interview. Our professional interviewers obtained verbal consent and used computer assisted telephone interviews (CATI) which took approximately 45 min.

### Survey instrument

All study materials had a readability level ≤8th grade, and are culturally sensitive and relevant (Bailey et al. 2000).

### Physical impairments

Participants were asked whether they had BC-related impairments that fall under the scope of PT practice (1 = Yes, 0 = No) to indicate presence of shoulder movement impairment, muscle weakness, pain, fatigue, skin numbness, and abnormal posture.

### Physical therapy utilization

We asked participants whether they utilized PT (1 = Yes; 0 = No) for each impairment reported.

### Participant characteristics

Race was measured with self-report of AA or white using binary responses (1 = Yes; 0 = No). We measured SES with; (1) formal education achieved (<9, 9–11 years, high school/GED, vocational/technical, some college/junior college, and college graduate or higher) (American Community Survey Design and Methodology 2014) and (2) annual household income (<$10 K, $10–20 K, $21–40 K, and >$40 k). We approximate the 2011 US Census Bureau official poverty threshold to represent “low income” ($22,811 for a family of four). Thus, our categories <$10 K and $10–$20 K represented “low-income” (Census Bureau 2011).

Number of years since BC diagnosis was calculated as the year of diagnosis subtracted from the interview date and represents length of survival—a modification of a raw survival calculation with the end-point as the interview date, not death (Cho et al. 2011).

Health insurance, a well-known determinant of health care access, was classified by insurance type (Medicare, Medicaid, private carrier, Champus, other, and no insurance) (Lukavsky and Sariego 2015).

Because the SCCS sample is unique in recruitment method (face-to-face interviews in community health centers and general public recruitment), we include this variable to control for any variation related to recruitment source.

### Medical history and comorbid conditions

We control for medical history and comorbid conditions because these contribute to overall health and, in some cases, could explain some variance in our outcome variables (Gallicchio et al. 2014). Using yes/no response choices, participants were asked whether they had high blood pressure, heart attack, diabetes mellitus, stroke, emphysema, depression, osteoarthritis, congestive heart failure, HIV/AIDS, memory problems, paralysis and menopause. These are the same comorbidities asked in the SCCS baseline questionnaire and the sum was used to represent comorbidity (Schou et al. 2012).

Body mass index (BMI) was calculated as self-reported current weight (kg) divided by height^2^ (m) from the most recent SCCS survey completed.

### Barriers to physical therapy utilization

We asked participants about patient, physician, health care system and financial barriers to health care utilization (Fradgley et al. 2015) revised to reflect PT. Patient barriers include: forgot to schedule/attend PT; fear of cancer recurrence/spread; too busy to attend PT; embarrassed; fear of discomfort/pain; lacks social support to attend PT; lacks knowledge of PT clinic location. Physician and health care system barriers include: doctor has not recommended PT; fear of prejudice or racism; inconvenient PT clinic hours; lack of child/elder care services to attend PT; attending PT interferes with spending time with family. Financial barriers include: high cost; lack of insurance coverage for PT; cannot afford to take time off from work to attend. We used yes (1)/no (0) responses and then summed the number of barriers (maximum of 16).

### Statistical analysis

We stratified our sample by race (AA or white) and annual household income to compare impairments and PT utilization. We present frequencies and percentages for categorical variables and means and standard deviations for continuous measures to characterize our participants in terms of sociodemographics, medical history/comorbidity, and prevalence of impairments. Differences by race were assessed with Chi square tests or Fisher’s exact tests for categorical variables and independent samples t-tests for continuous variables. Income differences were assessed with Mantel–Haenszel Chi square tests for ordinal variables, Cochran–Armitage trend tests for categorical variables and ANOVA for continuous variables. We used multiple logistic regression to adjust for potential confounders and present adjusted odds ratios with 95 % confidence intervals. Statistical significance was determined with P values <0.05 and all analyses were performed using SAS version 9.2 (SAS Institute, Cary, NC).

## Results

### Sample size

Of 1109 women reported having been diagnosed with BC prior to SCCS enrollment and not previously known to have died, we completed 577 interviews. Of non-participants, 337 could not be reached by telephone, 13 never had cancer, 68 refused, 65 could not be reached, and 49 died per National Death Index verification. Of the responding 577 BCS, those missing race (n = 42) or those not identified as non-Hispanic white or AA (n = 7) were excluded from race-specific analyses yielding a sample of 528 BCS for all analyses comparing racial groups. The sample size was reduced to 524 for income comparisons because 4 additional participants had missing data, refused to answer or did not know.

### Sample characteristics by race and income

On average, BCS had survived cancer for 12 years and were approximately 64 years of age at the time of interview. AAs were significantly younger than whites (62 vs. 65 years). Forty-seven percent reported incomes of $20 K or less, with significantly lower incomes among AAs than whites.

Whites were more likely than AAs to report long-term BC medication. Those with low income were more likely to have mastectomy and less likely to have had radiation therapy. More than half reported high blood pressure and 25 % had diabetes, with AAs more likely than whites to have these conditions (Table 1).

**Table 1:** Characteristics by race and income

Characteristic^a^	Overall N = 577	Race		P value^b^	Income				P value^c^
		White N = 266	AA N = 262		≤$10 k N = 119	$10,001–$20,000 N = 129	$20,001–$40,000 N = 137	≥$40,000 N = 139	
Age (years), mean	63.7	64.9	62.2	0.0002	63.3	64.6	64.7	61.2	0.0017
Education (%)									
<High school	17.7	15.5	17.9	0.79	37.3	19.8	11.0	0.0	<0.0001
High school	27.9	29.4	27.2		29.7	39.7	30.1	13.0	
Some College/vocational training	29.4	27.6	30.4		26.3	29.4	36.0	26.8	
College graduate	13.7	14.7	14.0		4.2	8.7	12.5	29.0	
Graduate school	11.4	12.8	10.5		2.5	9.6	9.6	31.2	
Missing/refuse	1.0	0.4	1.9		0.8	2.3	0.7	0.7	
No. of years since BC diagnosis, mean	12.6	12.7	12.5	0.74	13.4	13.4	12.5	11.3	0.073
BMI, mean	31.2	29.5	32.9	<0.0001	32.5	31.9	31.0	29.2	0.0014
% Income^c^									
<$10 K	22.7	29.0	33.3	<0.0001	–	–	–	–	–
$10 K–$20 K	24.6	24.7	24.7		–	–	–	–	–
$20 K–$40 K	26.2	30.5	22.4		–	–	–	–	–
>$40 K	26.5	33.6	19.6		–	–	–	–	–
Race^b^									
White	–	–	–	–	11.2	24.7	30.5	33.6	<0.0001
AA	–	–	–		33.3	24.7	22.4	19.6	
Enrollment location (%)									
Community health center	58.6	54.1	60.7	0.13	75.6	71.3	51.1	35.3	<0.0001
Public recruitment	41.4	45.9	39.3		24.4	28.7	48.9	64.8	
Insurance (%)*									
Medicaid	17.3	11.3	22.5	0.0006	52.1	14.1	5.8	0.7	<0.0001
Medicare	54.8	57.1	52.7	0.30	58.8	70.5	55.5	36.7	<0.0001
Private	53.5	60.2	47.7	0.0041	18.5	36.4	67.2	86.3	<0.0001
Champus	4.5	4.9	4.2	0.70	0.8	1.6	6.6	7.2	0.0036
Other	9.3	10.2	8.8	0.59	8.4	10.9	11.0	7.2	0.56
None	8.0	7.5	7.3	0.90	10.1	10.1	11.0	0.7	0.0021
Missing/refuse	6.8	0	0	–	52.1	14.1	5.8	0.7	<0.0001
BC treatment (%)									
Any surgery	98.4	98.5	98.9	0.72	99.2	100.0	97.8	97.8	0.18
Mastectomy	60.5	61.1	61.0	0.99	68.6	65.9	59.0	50.7	0.0014
Lumpectomy	46.4	48.9	43.2	0.20	33.1	41.9	50.0	58.8	<0.0001
Sentinel or axillary node dissection	58.7	62.9	55.9	0.11	64.9	52.4	53.4	65.2	0.47
Chemotherapy	47.2	45.9	49.4	0.41	46.6	45.0	46.7	52.5	0.25
Radiation	47.4	47.7	48.7	0.83	40.2	41.1	48.5	60.4	0.0002
Long term medication	47.3	52.3	43.5	0.044	38.1	45.3	53.3	49.6	0.072
No. of modalities mean (SD)	3.2 (1.4)	3.4 (1.5)	3.2 (1.3)	0.091	3.0 (1.2)	3.1 (1.4)	3.3 (1.4)	3.6 (1.4)	0.0042
High blood pressure (%)	53.9	39.8	68.3	<0.0001	71.4	60.9	50.4	37.0	<0.0001
Myocardial Infarct (%)	5.1	3.8	5.0	0.50	4.2	8.5	4.4	0.7	0.022
Diabetes (%)	25.8	16.2	34.0	<0.0001	37.0	33.3	19.0	13.7	<0.0001
Stroke (%)	4.5	4.1	4.6	0.80	7.6	8.5	0.7	2.2	0.0037
Hepatitis (%)	2.1	1.1	3.4	0.076	5.9	1.6	1.5	0.7	0.028
Emphysema (%)	8.4	8.6	8.1	0.81	17.6	7.0	6.6	4.3	0.0008
Depression (%)	23.0	25.2	21.4	0.30	40.3	27.1	16.1	12.9	<0.0001
Osteoarthritis (%)	57.8	56.0	60.7	0.28	77.3	53.5	61.3	43.2	<0.0001
Menopause (%)	96.0	98.5	93.9	0.0056	91.6	99.2	97.8	95.0	<0.0001

^a^Congestive heart failure, HIV/AIDS, memory problems, paralysis, were excluded because they were not significant

^b^Comparisons by race are independent samples *t* tests (continuous variables) and Chi square tests (categorical variables)

^c^Differences between income groups are from ANOVA tests (continuous variables) and Chi square tests (categorical variables)

* Participants selected all applicable insurance types and combinations reported are: Medicaid + Medicare (n = 43), Medicaid + Medicare + Other (n = 13), Medicaid + Other (n = 8), and Medicare + Other (n = 152)

We also examined whether participants differed from BCS that did not enroll in our study in terms of these demographic characteristics. Participants tended to be older (mean age at cohort entry of 59.3 vs. 56.7 years and mean age at BC diagnosis as 50.5 vs. 47.4 years) and more often white (52 vs. 42 %). The interviewees also tended to be of higher income at SCCS cohort entry, with 44 vs. 22 % having household incomes over $20,000. On all other variables, participants did not differ from those who did not participate in the study. While these differences require caution in the generalizing the findings, comparisons made among the participants are internally valid because all BCS completed the same questionnaire following identical methods.

### Impairments by race and income

Impairment prevalence was highest for skin numbness at 57 % followed by fatigue (55 %), pain (43 %), muscle weakness (38 %), shoulder movement impairment (31 %) and postural abnormality (15 %). There were no significant differences in impairment prevalence by race (Table 2).

**Table 2:** Percent impairments and PT by race

Impairment	% impairment			% PT use		
	AA %^a^	White %^a^	Adj. OR^b,c^ (95 % CI)	AA %^a^	White %^a^	Adj. OR^b,c^ (95 % CI)
Shoulder	34.7	29.8	0.97 (0.63, 1.50)	42.9	25.3	2.56 (1.11, 5.90)
Muscle weakness	39.6	38.8	0.65 (0.42, 1.00)	27.2	18.6	2.43 (1.00, 5.87)
Pain	47.3	42.1	0.85 (0.56, 1.30)	23.4	12.5	3.16 (1.33, 7.50)
Fatigue	56.5	58.6	0.64 (0.41, 0.99)	5.4	5.8	1.00 (0.32, 3.19)
Skin numbness	59.9	58.6	0.83 (0.54, 1.28)	9.6	5.0	2.27 (0.77, 6.69)
Postural abnormality	16.2	15.4	0.78 (0.44, 1.36)	14.3	12.2	5.46 (0.52, 57.43)

^a^% in race group where the denominators are: for AA, n = 262; whites n = 266

^b^White is reference group

^c^ORs adjusted for age, income, recruitment location, years since BC diagnosis, comorbidity, BMI, BC treatment

The average number of impairments (among 6 queried) was 2.5 (SD = 1.8), but higher for those with lowest-incomes (2.8) versus higher incomes (2.3). Those with household income below $10,000 reported 0.8 more impairments than those with incomes over $40,000 (P = 0.006). Impairment prevalences were higher among those in the lowest income categories—significantly so for pain and muscle weakness (Table 3). Barriers to care were not significantly associated with impairments by race or income.

**Table 3:** Percent impairments and PT by income

Impairment	Income	% impairment		% reporting PT by impairment	
		%^a^	Adj. OR (95 % CI)^b^	%	Adj. OR (95 % CI)^b^
Shoulder					
	<$10 K	42.0	1.48 (0.68–3.27)	26.0	1.05 (0.25–4.39)
	$10 K–$20 K	31.0	0.84 (0.43–1.63)	47.5	2.70 (0.81–8.95)
	$20 K–$40 K	27.2	0.88 (0.49–1.59)	24.3	0.61 (0.19–1.93)
	>$40 K (ref)	28.8	1 (ref)	40.0	1 (ref)
Muscle weakness	<$10 K	54.6	2.21 (1.00–4.86)	13.9	0.47 (0.09–2.33)
	$10 K–$20 K	42.2	1.44 (0.74–2.77)	40.7	3.04 (0.82–11.32)
	$20 K–$40 K	36.8	1.34 (0.75–2.41)	18.0	1.07 (0.29–3.94)
	>$40 K (ref)	27.9	1 (ref)	18.4	1 (ref)
Pain	<$10 K	58.0	2.66 (1.23–5.76)	11.6	0.40 (0.08–1.93)
	$10 K–$20 K	40.3	1.01 (0.54–1.89)	32.7	1.94 (0.56–6.73)
	$20 K–$40 K	45.3	1.53 (0.89–2.62)	16.1	1.01 (0.33–3.16)
	>$40 K (ref)	38.1	1 (ref)	15.1	1 (ref)
Fatigue	<$10 K	64.7	1.49 (0.67–3.33)	5.2	2.34 (0.16–35.13)
	$10 K–$20 K	57.8	0.94 (0.50–1.79)	13.5	6.52 (0.64–66.02)
	$20 K–$40 K	54.0	1.01 (0.58–1.75)	2.7	1.98 (0.16–23.89)
	>$40 K (ref)	54.4	1 (ref)	1.3	1 (ref)
Skin numbness	<$10 K	63.9	1.71 (0.77–3.81)	7.9	2.46 (0.34–17.92)
	$10 K–$20 K	55.8	0.78 (0.42–1.46)	12.5	4.27 (0.90–20.41)
	$20 K–$40 K	59.1	1.05 (0.60–1.83)	4.9	1.23 (0.28–5.51)
	>$40 K (ref)	62.6	1 (ref)	5.8	1 (ref)
Postural abnormality	<$10 K	26.3	1.88 (0.67–5.31)	16.1	1.57 (0.02–103.29)
	$10 K–$20 K	17.3	1.52 (0.63–3.71)	13.6	0.42 (0.02–8.26)
	$20 K–$40 K	11.0	1.03 (0.45–2.39)	13.3	1.90 (0.07–51.69)
	>$40 K (ref)	10.8	1 (ref)	13.3	1 (ref)

^a^% income category where denominators are: ≤$10 k, n = 119; $10,001–$20,000, n = 129; $20,001–$40,000, n = 137; ≥$40,000, n = 139

^b^Odds ratios adjusted for age, race, recruitment location, years since BC diagnosis, comorbidity, BMI, BC treatment types

### PT utilization by race and income

Those with shoulder movement impairment were most likely to utilize PT (33 %), followed by muscle weakness (22 %), pain (18 %), postural abnormality (14 %), skin numbness (8 %), and fatigue (5 %) (Table 2). AAs were significantly more likely than whites to utilize PT for shoulder movement impairment and pain. No clear or significant associations between PT utilization and income were apparent.

## Discussion

Low-income was associated with greater impairment prevalence, yet low-income BCS did not report higher PT use. Our study is the first to report on differences of impairments and PT utilization for BCS by race and income. Prior research indicates that AA BCS report higher prevalence of pain, fatigue, and lower overall physical and functional quality of life (Green et al. 2003; Paskett et al. 2008), but among the BCS we interviewed race was inconsistently associated with impairments. Most research on BCS involves white middle-income women with health insurance. Our study has the unique advantage of examining impairments in a large, diverse sample of BCS of all income levels.

Our research supports work that shows those with lower income have significantly lower physical functioning when compared to higher income whites and minorities (Braithwaite et al. 2010). A cross-sectional, observational study of BCS found that 36–59 % had shoulder range of motion restrictions (Cheville et al. 2008). Others found that BCS had significantly more shoulder limitations when compared to controls (Harrington et al. 2011). Our findings highlight the importance of shoulder range of motion limitation and suggest more of a need to address impairments—especially for those with low incomes.

Poorly controlled pain contributes to compensatory patterns of overuse, poor posture, and faulty biomechanics (Norkin and Levangie 1992). Other studies report that AAs may have higher levels of cancer-related pain (Green et al. 2003) yet we found significant differences in pain by income—not race.

Fatigue affects 9–39 % of BC (American Community Survey Design and Methodology 2014; Palmer et al. 2013) and is associated with disability and health care utilization (Servaes et al. 2007). One longitudinal study of 252 BCS reports fatigue affecting 31 % right after treatment declining to 6 % by the end of the first post-treatment year (Green et al. 2003). The majority of our survivors reported fatigue—a substantial finding because our BCS were long-term survivors. Another longitudinal study of 244 long-term BCS found no racial differences in fatigue, pain and other side effects, but they did not make comparisons by income (Gill et al. 2004). Our research shows significant differences by income for pain and utilization of PT to treat pain but not for fatigue.

Our study suggests that PT may be under-utilized for these impairments despite insurance status. The significantly higher use of PT by AAs was unexpected. Since we studied the presence/absence of impairments, not severity, it is possible that severe impairment might be more likely to trigger a referral to PT. In our sample, AAs were more likely to utilize PT if they had complaints of shoulder impairment, muscle weakness or pain. The total number of barriers to PT was not significantly associated with PT utilization—a surprising finding. While not all BCS need PT, our study suggests that impairments persist and the need for referral to PT may also persist.

Recent rehabilitation services documentation for Medicare patients mandate the use of patient reported outcomes such as those examined in this study. It is possible that some BCS may have issues with recall and attribution of impairments to cancer rather than an alternative medical diagnosis. Since our subjects were drawn from many different states, verification of self-reports was logistically impossible because it would have involved hundreds of hospitals, outpatient clinics and internal review boards.

Although this research included high proportions of low-income BCS (an advantage), the relatively few individuals with higher incomes precluded assessing income effects across upper-middle and higher income ranges. While BC is not rare, we studied a large proportion of minority and poor BCS which is rare and has allowed us to consider three key social determinants—race, income, and insurance.

Our results could also be partially explained by residence differences. For example, rural residents are more likely than urban residents to forgo medical and dental care after cancer (Palmer et al. 2013), travel farther for care and have mastectomy (Meilleur et al. 2013). Areas with higher versus lower health care spending also have higher rates of recommended and preferred health care (Keating et al. 2012). Future research can include these comparisons by taking advantage of hierarchical statistical modeling.

Our results may reflect the effects of reduced physical activity among breast cancer survivors. While exercise has many benefits including cancer control and prevention (Courneya et al. 2014) exercise is not part of survivorship care (Phillips et al. 2014).

Our study suggests that disparities exist among BCS for impairments that fall in the scope of practice for physical therapists. The “surveillance” model (Campbell et al. 2012) tracks signs and symptoms indicative of BC-related impairments, but ownership of surveillance is not clear nor tested with population-based samples. However, rehabilitation models exist in all medical systems that can be leveraged to improve BC-related impairment management. Rehabilitation is standard for those with total joint replacement and significantly improves physical and functional ability (Desmeules et al. 2013) self-efficacy, (Lane-Carlson and Kumar 2012) pain, (Niu et al. 2011) and reduces hospital length of stay, (Mertes et al. 2013) post-operative complications, (Husni et al. 2010) and readmission. (Gooch et al. 2012) The inclusion of PT at the beginning of treatment planning could have a similar effect in the BCS population, especially for those with low-incomes who seem to have the most BC-related impairments. Comparative effectiveness trials could compare referral-based versus rehabilitation models of care delivery.

## Conclusions

Impairment burden is high among BCS but especially the poor—precisely those who are least able to afford it. The high prevalence of impairments and low PT utilization suggest that an unmet need for rehabilitation services may exist. Persistent impairments can interfere with the ability to return to work (Clarke et al. 2011) and if untreated may lead to disability. (Goldstein et al. 2012) Our detection of low PT utilization suggests that strategies to enhance opportunities for PT may lighten BC-related impairment burden.
